# *Mycobacterial* PPE36 Modulates Host Inflammation by Promoting E3 Ligase Smurf1-Mediated MyD88 Degradation

**DOI:** 10.3389/fimmu.2022.690667

**Published:** 2022-02-14

**Authors:** Zhangli Peng, Yan Yue, Sidong Xiong

**Affiliations:** Jiangsu Key Laboratory of Infection and Immunity, Institutes of Biology and Medical Sciences, Soochow University, Suzhou, China

**Keywords:** *Mycobacteria tuberculosis*, PPE36, Smurf1, MyD88, degradation

## Abstract

*Mycobacterium tuberculosis* (Mtb) PPE36, a cell-wall-associated protein, is highly specific and conserved for the Mtb complex group. Although PPE36 has been proven essential for iron utilization, little is known about it in regulating host immune responses. Here we exhibited that PPE36 was preferentially enriched in Mtb virulent strains and could efficiently inhibit host inflammatory responses and increase bacterial loads in infected macrophages and mice. In exploring the underlying mechanisms, we found that PPE36 could robustly inhibit the activation of inflammatory NF-κB and MAPK (Erk, p38, and Jnk) pathways by promoting E3 ligase Smurf1-mediated ubiquitination and proteasomal degradation of MyD88 protein. Our research revealed a previously unknown function of PPE36 on modulating host immune responses and provided some clues to the development of novel tuberculosis treatment strategies based on immune regulation.

## Introduction

Tuberculosis is a global health emergency with about 10 million new TB cases and 1.2 million deaths in 2019 (1). However, tuberculosis death in many countries appeared rapidly declined over the past decades, but not fast enough to reach the 2020 milestone of a 20% reduction between 2015 and 2020. The cumulative reduction from 2015 to 2019 was 9%, including a reduction of 2.3% between 2018 and 2019 (1). In addition, inoculation of the only widely used tuberculosis vaccine Bacillus Calmette-Guérin (BCG) could not be effective for all populations; therefore, the global burden of tuberculosis remains substantial.

As an extremely successful pathogen, *Mycobacterium tuberculosis* (Mtb) has coevolved with human beings for thousands of years and developed diverse mechanisms to establish latent, progressive, or persistent infections in fully immunocompetent hosts. Host macrophages are the initial and primary targets of Mtb infection. In there, they reside and persist by a variety of immune evasion strategies, including hiding from pattern recognition receptor (PRR) recognition (2), preventing T cell responses by downregulating major histocompatibility complex (MHC) molecules (3), and evading macrophage killing systems (3). In addition, the genomic plasticity of Mtb might also be partially responsible for the antigenic variations of diverse strains and the variable protective efficacy of diverse BCG strains (4).

Actually, some important comparative analyses have shown us the panoramic views of genome variations among BCG vaccine strains (4, 5). Wan et al. (6) showed 43 common proteins between 13 BCG strains. Most of them belonged to PE and PPE protein families with unknown functions. PE and PPE are two families of glycine-rich proteins with a repetitive structure; their nomination is derived from the conserved N-terminal Pro-Glu (PE) and Pro-Pro-Glu (PPE) motifs. In addition to representing a principal source of antigenic variation, more and more PE/PPE proteins exhibited abilities to modulate host immune responses (7).

In this study, we focused on exploring the potential role of PPE36 in the interaction between Mtb and macrophages. PPE36 is a 27-kDa cell-wall-associated protein and limitedly expressed among the Mtb complex group (including notably *Mycobacterium tuberculosis*, *Mycobacterium africanum*, *Mycobacterium bovis*) (8–10). A recent study revealed that PPE36 was essential for the iron acquisition from heme by Mtb (11). Interestingly, there seemed to be a paradox about PPE36. On the one hand, it is dispensable for mycobacteria survival and growth (12); on the other hand, it is ubiquitous in various bacterial strains (6, 13). We hypothesized that PPE36 might have other functions beneficial for Mtb survival and/or infection. In fact, several cell-membrane-associated PPE proteins (such as PPE18 and PPE34) have shown to interact with host immune systems, like binding TLR2 and decreasing MHC molecules (14, 15). More importantly, PPE37, another Mtb PPE member facilitating iron utilization just like PPE36, was found to be able to decrease pro-inflammatory cytokine production in macrophages (16, 17). All these works further prompted us to explore the possible influence of PPE36 on Mtb-induced macrophage immune responses.

In this study, we found that PPE36 could promote the ubiquitination and degradation of the host MyD88 protein by facilitating the interaction of MyD88 with the E3 ligase Smurf1 and then suppress the subsequent NF-κB as well as MAPK (Erk, p38, and Jnk) pathways, which led to the dampened host inflammatory responses and increased bacterial loads in macrophages. Our findings revealed a previously unknown immune modulation function of PPE36. It will help us better understand the complicated immune evasion mechanisms of Mtb and may also provide some clues to the development of novel preventive and therapeutic strategies against tuberculosis based on PPE proteins.

## Materials and Methods

### Mycobacterium Strains and cDNAs of Clinical Isolates

*M. bovis* BCG was obtained from the Shanghai Institutes for Biological Sciences. The BCG ΔPPE36 strain was purchased from Gene Optimal Inc. (Shanghai, China). WT BCG and BCG ΔPPE36 strains were grown at 37°C on Middlebrook 7H10 agar (BD Diagnostic Systems, Hunt Valley, MD, USA) supplemented with 10% ADC, or in Middlebrook 7H9 broth medium (BD Diagnostic Systems) supplemented with 10% OADC, 0.5% glycerol, and 0.05% Tween-80. Herein, *M. smegmatis* strains included WT *M. smegmatis* (WT *M.smeg*) and PPE36-overexpressing *M. smegmatis* (PPE36-*M.smeg*). PPE36-*M.smeg* was prepared as transfecting the plasmid pMV261-PPE36 into WT *M.smeg* by electroporation according to the standard procedure. The cDNAs of H37Rv and clinical isolates were obtained from the Affiliated Hospital of Zunyi Medical University in China.

### Cells, Plasmids, and Antibodies

HEK293T cells and murine macrophage cell line RAW264.7 were cultured in DMEM medium (HyClone, Logan, UT, USA) supplemented with 10% fetal bovine serum (HyClone), 0.1 mg/ml streptomycin, and 100 U/ml penicillin at 37°C with 5% CO_2_. Bone marrow-derived macrophages (BMDMs) were prepared as previously described (18). RAW264.7 cells stably transfected with retrovirus carrying pMSCV-eGFP or pMSCV-eGFP-PPE36 were named as RAW-Vector and RAW-PPE36 cells, respectively.

The mammalian expression plasmids pPPE36-Flag, pPPE36-Myc, and pMyD88-HA were constructed in our laboratory; plasmids pRacL61, pTRAF6, pTAB1, pTAB2, pTAB3, and pTAK1 were provided by Prof. Cuihua Liu (Chinese Academy of Sciences, China); plasmids encoding Smurf1-Myc, p-Ubiquitin (Ub)-Flag, pUb-His, pUb-His (K48), pUb-His (K63), and Smurf1 shRNA (sh-Smurf1) were provided by Prof. Hui Zheng (Soochow University, China). The dual-luciferase reporter assay vectors pNF-kB-luc, pAP1-luc, and pRL-TK were purchased from Clontech (Mountain View, CA, USA).

Primary antibodies against GAPDH, p-p65, p65, p-IκBα, IκBα, Tubulin, p-Jnk, Jnk, p-Erk1/2, Erk1/2, p-p38, and p38 (Cell Signaling Technology) were used for Western blot assays. Antibodies against MyD88, HA, Myc, Ubiquitin, Smurf1 (Cell Signaling Technology, Danvers, MA, USA), and Flag (Sigma) were used for immunoprecipitation assays. HRP-conjugated anti-mouse or anti-rabbit secondary antibody was purchased from SouthernBiotech (Birmingham, AL, USA).

### Quantification of PPE36 Expression by Real-Time PCR

The total RNAs were extracted from various mycobacterium strains and transcribed into cDNAs as previously described (19), and PPE36 expressions were quantified by absolute quantification real-time PCR using the standard curve method (20). Standard curves were generated using a dilution series of 10^10^ to 10^3^ copies per microliter of the linearizing pPPE36-Flag as a template. Using the average molecular weight of the product and Avogadro’s constant, the number of copies per unit volume was calculated as described previously (20–22).

### GeneXpert Assays

GeneXpert assays were used to determine bacterial loads in the clinical sputum and BALF samples as described (23). In brief, 1 ml sputum or 200 μl BALF decontaminated samples was added into 2 ml of sample reagent and transferred into a test cartridge. The cartridge then was inserted into the test platform of the GeneXpert instrument. Threshold cycle (CT) values for each of the five rpoB probes were recorded and used to estimate the bacterial loads. A CT value of 40 was assigned if GeneXpert was negative for Mtb detection.

### Mycobacterium Infection

Murine macrophage cell line RAW264.7 or BMDMs were infected with WT BCG or BCG ΔPPE36 at a multiplicity of infection (MOI) of 10, and then cells were harvested at different time points in the follow-up experiments.

Six-to-eight-week-old C57BL/6 mice were purchased from Shanghai SLAC Laboratory Animal Co., Ltd. (Shanghai, China) and housed using standard humane animal husbandry protocols at Soochow University. All animal experiments were approved by the Institutional Animal Care and Use Committees of Soochow University. Mice were intranasally infected with 1 × 10^7^ colony-forming units (CFU) of WT BCG or BCG ΔPPE36 and sacrificed at 7, 14, 21, or 28 days postinfection.

### Colony-Forming Unit Counting

BMDMs cells were seeded in 6-well plates, then were infected with WT BCG or BCG △PPE36 at a multiplicity of infection (MOI) of 10 for 4 h. Afterward, cells were washed with fresh medium and treated with 200 μg/ml amikacin for 1 h to kill extracellular bacteria. Fresh medium containing amikacin (20 μg/ml) was then used for the following 24-h incubation. Then, infected cells were lysed with 1 ml sterile water containing 0.05% Triton X-100. For CFU assay, 50 μl cell lysates was plated on the 7H10 medium containing hygromycin and cultured for 4 weeks at 37°C.

CFU counting was applied to determine the bacterial loads of murine lung tissues. Homogenized lung tissues were diluted with PBS. Moreover, 50 μl of cell lysates or tissue homogenates was added to 7H10 plates and cultured for 4 weeks, and then CFU counting was performed.

### Acid Fast Staining and Lung Injury Evaluation

Lung tissues were fixed with 4% (v/v) paraformaldehyde, embedded in paraffin for slicing, and then subjected to hematoxylin and eosin staining. The Ziehl–Neelsen acid fast staining was performed to analyze the bacterial load in the lung sections as previously described (24). The sections were analyzed by lung injury score to score lung inflammation and damage (25). Microscopic scoring criteria of lung injury were graded from 0 to 4: Grade 0: normal lung morphology; Grade 1: mild intra-alveolar edema and mild inflammatory cell infiltration; Grade 2: moderate intra-alveolar edema and moderate inflammatory cell infiltration; Grade 3: severe alveolar edema, severe inflammatory cell infiltration, and focal hemorrhage; and Grade 4: disseminated inflammatory cell infiltration and destruction in alveolar structure.

### Luciferase Reporter Assays

For detecting NF-κB activity detection, HEK293T cells were co-transfected with pNF-κB-luc (1 μg) and pRL-TK (50 ng) plasmids in the presence of pPPE36 (1 μg) or control plasmid (1 μg) for 24 h using Lipofectamine 2000 reagent (Invitrogen, Carlsbad, CA, USA). Then, cells were treated with TNF-α (20 ng/ml) for 12 h. For detecting Jnk and p38 activation, HEK293T cells were co-transfected with pRacL61 (1 μg), pAP1-luc (1 μg), and pRL-TK (50 ng) in the presence of PPE36-Flag (1 μg) or control plasmid (1 μg) for 36 h. Cells were lysed and subjected to luciferase activity detection according to manufacturer instructions (Promega, Madison, WI, USA). All reactions were performed in triplicate.

### Western Blot Analysis

Cells lysates were fractionated by 10% SDS-PAGE, transferred onto PVDF membranes, and incubated with primary antibodies against tubulin, GAPDH, PPE36, or molecules associated with NF-κB and AP1 signaling pathways. After washing with PBST three times, membranes were further incubated with the appropriate HRP-conjugated anti-rabbit or anti-mouse secondary antibody. Signals were detected by enhanced chemiluminescence (ECL) by Amersham Imager 600 (GE) and quantified with the ImageJ software.

### Immunoprecipitation and Ubiquitination Assays

HEK293T cells were co-transfected with pMyD88-HA (0.5 μg), pPPE36-Flag (1.5 μg), pSmurf1-Myc (1.5 μg), and pUb-Flag (50 ng) for 36 h, and then cell lysates were prepared and immunoprecipitated with anti-HA or anti-MyD88 beads. To detect the K48- or K63-linked ubiquitination levels of MyD88 protein, 293T cells were co-transfected with pPPE36-Flag (1.5 μg), pMyD88-HA (0.5 μg) in combination with pUb-His (50 ng), pUb-His (K48) (50 ng), or pUb-His (K63)(50 ng) for 36 h. Cell lysates were prepared and immunoprecipitated with anti-HA beads. The precipitates were then immunoblotted with anti-Ub, anti-Smurf1, anti-Myc, anti-HA, anti-Flag, or anti-MyD88 antibodies.

### Statistical Analysis

All data were expressed as mean ± SEM. Statistical differences in 2 groups or more than 2 groups were respectively assessed by Student’s t test or one-way ANOVA followed by Bonferroni test using GraphPad Prism version 6.0 (GraphPad Software Incorporated, San Diego, CA, USA). p values less than 0.05 were considered to be significant.

## Results

### PPE36 Was Predominantly Enriched in the Virulent but Not Attenuated Mycobacteria

**Figure 1:**
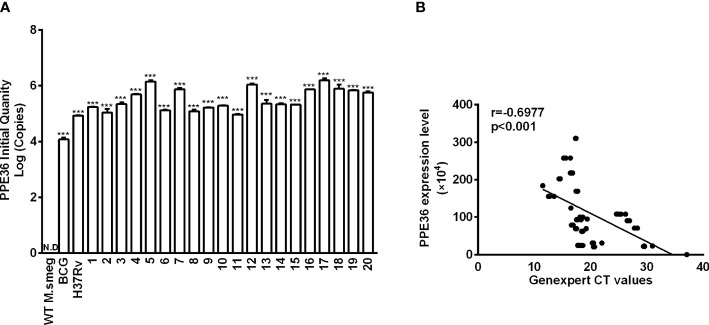
PPE36 expression was predominantly enriched in the virulent mycobacteria. **(A)** PPE36 expression in WT *M. smeg*, BCG, H37Rv, and clinical isolates (n = 20) was quantified by real-time PCR assays. N.D, no detection. **(B)** Correlation of PPE36 expression in clinical isolates with the bacterial loads in clinical samples. ***p <0.001.

PPE36 expression in WT M.smeg strain, BCG strain, Mtb H37Rv strain, or 20 Mtb clinical isolates was detected by the absolute quantification real-time PCR assays. It was found that PPE36 was much more enriched in the virulent H37Rv strain than the attenuated BCG strain, and it was further significantly increased in almost 20 clinical isolates (**Figure 1A**), indicating that similar to other PPE family members (26), PPE36 is also preferentially expressed in the virulent mycobacteria.

Next, we explored the correlation of PPE36 expression in the 20 clinical isolates with the bacterial loads in clinical sputum and BALF samples. Herein, we applied for GeneXpert PCR assays to detect sputum or BALF Mtb and used the CT value to indirectly reflect the sputum bacterial loads, as the more bacterial load in sputum, the lower the GeneXpert CT value will be. Of interest, we found that PPE36 expression was significantly and negatively correlated with the GeneXpert CT values (r = -0.6977) (**Figure 1B**), which meant that PPE36 expression in the clinical isolates was closely and positively correlated with the bacterial loads in the clinical isolates. These data suggested that PPE36 might play an important role in Mtb infection and tuberculosis disease process.

### PPE36 Suppressed Macrophage Inflammatory Cytokine Production

**Figure 2:**
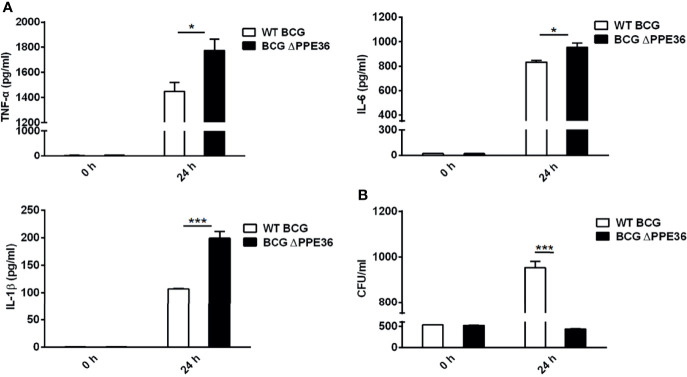
PPE36 suppressed macrophage inflammatory cytokine production. **(A)** Levels of TNF-α, IL-6, and IL-1β in the WT BCG- or BCG △PPE36-infected BMDMs. **(B)** Bacterial loads in the WT BCG- or BCG ΔPPE36-infected BMDMs. *p < 0.05, ***p <0.001.

Considering that the amino acid motif of BCG-derived PPE36 is identical to that of Mtb PPE36, herein we applied PPE36-depleted BCG (BCG ΔPPE36) to explore the potential impact of PPE36 on the macrophage inflammatory responses. As shown in **Figures 2A, B**, a dramatically increased production of inflammatory cytokines (p < 0.05) and a robustly lower intracellular bacterial load (p < 0.05) were evidenced in the BCG ΔPPE36-infected cells compared with those of the WT BCG-infected cells. These data indicated that PPE36 could facilitate intracellular mycobacterium survival by inhibiting inflammatory cytokine production.

### PPE36 Depletion Led to the Increased Inflammation and Decreased Bacterial Loads in the Lung Tissues of Mycobacterium-Infected Mice

**Figure 3:**
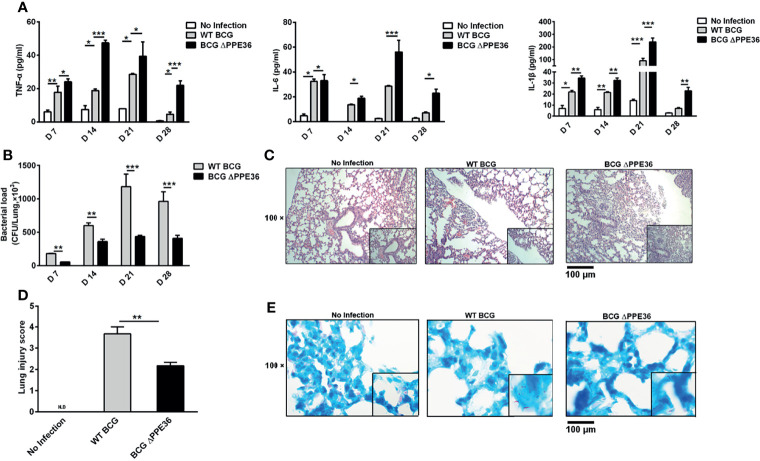
PPE36 depletion led to the increased inflammation and decreased bacterial loads in the lungs of BCG infected mice. **(A)** Levels of inflammatory cytokines in lung tissues of mice infected with WT BCG or BCG ΔPPE36. **(B)** Bacterial loads in the lung tissues of infected mice. **(C)** Pathological observation of lung tissues of infected mice. **(D)** Lung injury scores of infected mice at day 28 postinfection. N.D, not detected. **(E)** Lung tissue sections from WT mice infected with WT BCG or BCG ΔPPE36 were analyzed with acid-fast staining. *p < 0.05, **p < 0.01, ***p <0.001.

Mice were intranasally infected with WT BCG or BCG ΔPPE36 for various periods, and the levels of lung inflammatory cytokines were monitored. As shown in **Figure 3A**, PPE36 depletion appeared to increase the lung IL-1 β level at as early as day 7 postinfection, and this trend was even more pronounced on day 14, when the lung IL-6 and TNF-α levels also began increasing. This obvious gap maintained until day 28 postinfection. Meanwhile, decreased lung bacterial loads by bacterial colony-forming counting and acid-fast staining (**Figures 3B, E**) and the improved lung pathological observations (**Figures 3C, D**) were also observed in the BCG ΔPPE36 group compared with those in the control WT BCG group.

### Suppressive Effect of PPE36 on Macrophage Inflammation Relied on Dampening the NF-κB/MAKP Pathway Activation *via* Promoting MyD88 Degradation

**Figure 4:**
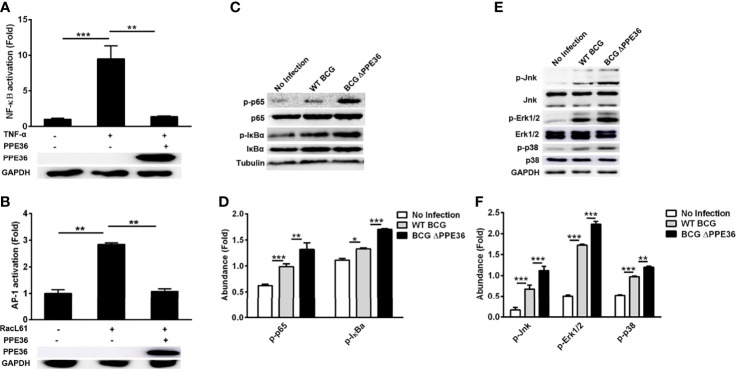
PPE36 inhibited NF-κB and MAPK activation. **(A)** Luciferase assays of NF-κB activation in PPE36-overexpressing 293T cells after treating with TNF-α (20 ng/ml). **(B)** Luciferase assays of AP1 activation in PPE36-overexpressing 293T cells after transfecting pRacL61. **(C, D)** Western blot analysis of p-p65, p65, p-IκBα, and IκBα expression in WT BCG- or BCG ΔPPE36-infected RAW264.7 cells. **(E, F)** Western blot analysis of p-Jnk, Jnk, p-Erk1/2, Erk1/2, p-p38, and p38 expression in WT BCG- or BCG ΔPPE36-infected RAW264.7 cells. Densitometry quantification of Western blot results were analyzed by ImageJ. *p < 0.05, **p < 0.01, ***p < 0.001.

To explore the modulation mechanisms of PPE36 on macrophage inflammation, we evaluated its influence on the activation of the NF-κB and MAPK pathways using a dual-luciferase reporter system, which contained a pNF-κB-Luc or pAP1-Luc vector and an internal control Renilla pRL-TK luciferase vector. As shown in **Figures 4A, B**, PPE36 overexpression could robustly reduce the NF-κB activity stimulated by TNF-α and AP-1 activity stimulated by RacL61. The declines were as high as about 85% and 60%, respectively. These data were further confirmed by Western blot results (**Figures 4C–F**). Taken together, the above evidence displayed that PPE36 simultaneously dampened NF-κB and MAPK activation, suggesting that PPE36 might act on the common upstream molecules of these two pathways.

**Figure 5:**
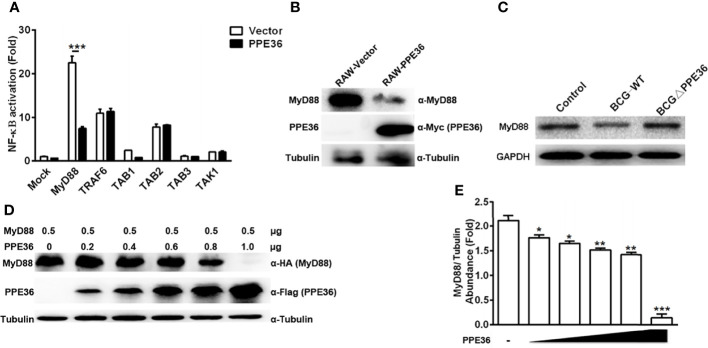
PPE36 facilitated host MyD88 protein degradation. **(A)** Luciferase assay of NF-κB activation in HEK293T cells co-transfected with pPPE36 and plasmids encoding various signaling molecules (TAK1, TAB1, TAB2, TAB3, TRAF6, and MyD88) respectively. **(B)** MyD88 expression in PPE36-stably expressing RAW264.7 cells (RAW-PPE36). **(C)** MyD88 expression in the WT BCG- or BCG ΔPPE36-infected macrophages. **(D, E)** MyD88 expression in the HEK293T cell line transfected with different doses of pPPE36-Flag. *p < 0.05, **p < 0.01, ***p <0.001.

Next, by co-transfecting plasmids encoding PPE36 and various signaling molecules (TAK1, TAB1, TAB2, TAB3, TRAF6, and MyD88), we tried to identify the potential PPE36 target by evaluating NF-κB activity with dual-luciferase reporter assays. In contrast to the hardly changed NF-kB activation in cells overexpressing other signaling molecules, a robust reduced NF-κB activity was observed in MyD88-overexpressing cells (**Figure 5A**), indicating that MyD88 was most likely to be the PPE36 target. Consistently, we also observed much less expression of MyD88 in the PPE36-stably expressing macrophages (**Figure 5B**), while an obvious decreased MyD88 expression was evidenced in BCG-infected macrophages, and this decrease was deprived by PPE36 depletion, further confirming that PPE36 could reduce MyD88 expression (**Figure 5C**). To further confirm that PPE36 could reduce the MyD88 protein abundance, we transfected different doses of pPPE36-Flag plasmid into MyD88-overexpressing 293T cells and then detected the MyD88 expression by Western blot. As shown in **Figures 5D, E**, PPE36 potentially attenuated MyD88 expression in a dose-dependent way. All these data indicated that PPE36 could inhibit macrophage inflammation *via* reducing MyD88 abundance.

### PPE36 Promoted E3 Ligase Smurf1-Mediated, K48-Linked Poly-Ubiquitination and Degradation of MyD88 Protein

**Figure 6:**
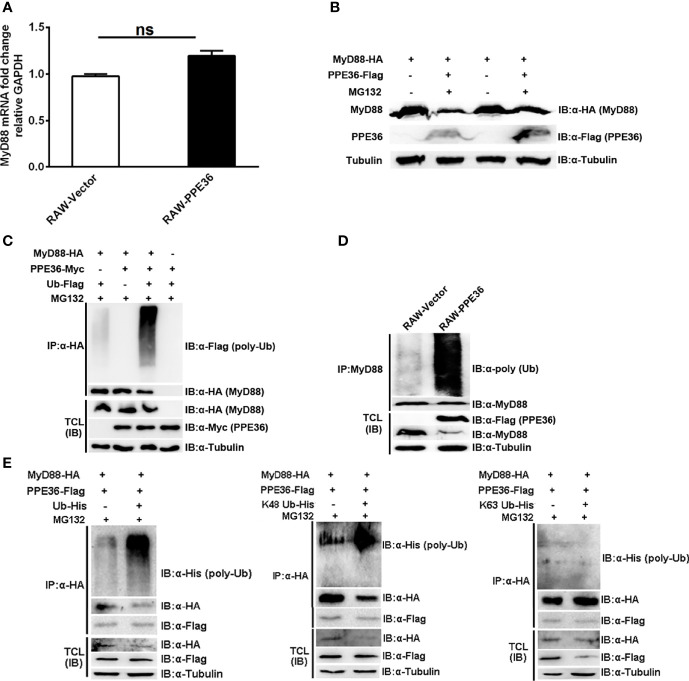
PPE36 promoted MyD88 ubiquitination. **(A)** MyD88 mRNA expression in PPE36-stably expressing RAW264.7 cells (RAW-PPE36). **(B)** MyD88 protein expression in HEK293T cells which were co-transfected with pMyD88-HA and pPPE36-Flag in the presence or absence of proteasome inhibitor MG132. **(C)** Ubiquitination of MyD88 protein in HEK293T cells co-transfected with the indicated combinations of plasmids. **(D)** Ubiquitination of MyD88 protein in RAW-PPE36 cells. **(E)** K48-linked and K63-linked ubiquitination of MyD88 protein in HEK293T cells co-transfected with the indicated combinations of plasmids. TCL, total cell lysates; IP, immunoprecipitation; IB, immunoblotting. ns, no significant.

Protein abundance depends on the dynamic balance between its synthesis and degradation. Herein we found that PPE36 hardly influenced MyD88 production at the transcription level (**Figure 6A**), suggesting that the post-translation degradation might be changed. This deduction was further supported by the increased MyD88 abundance in PPE36-overexpressing cells in the presence of proteasome inhibitor MG132 (**Figure 6B**), indicating that the ubiquitin (Ub)-protease degradation system was involved in PPE36-caused MyD88 reduction. In line with this, a much more intense ubiquitinated MyD88 ladder was evidenced in the PPE36-overexpressing 293T cells (**Figure 6C**) and macrophages (**Figure 6D**) compared with the control groups. These data exhibited that PPE36 could facilitate MyD88 ubiquitination and degradation. To determine the role of K48-linked and K63-linked ubiquitination in the PPE36-promoted MyD88 degradation, 293T cells were co-transfected with pPPE36-Flag and pMyD88-HA in combination with pUb-His, pUb-His (K48), or pUb-His (K63). As shown in **Figure 6E**, PPE36 could significantly increase K48-linked but could hardly influence the K63-linked poly-ubiquitination on MyD88 protein. These data indicated that PPE36 promoted MyD88 degradation by increasing K48-linked poly-ubiquitination.

**Figure 7:**
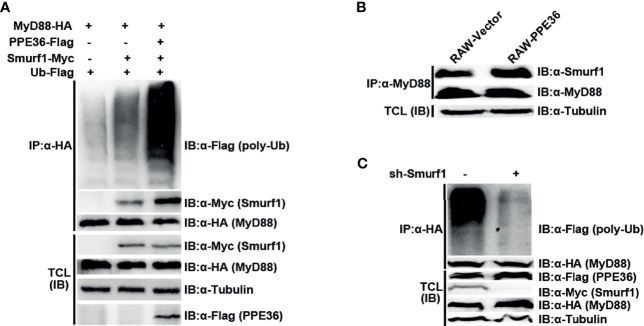
PPE36 promoted Smurf1-mediated ubiquitination and degradation of MyD88 protein. **(A)** Ubiquitination of MyD88 protein and the interaction of MyD88 with E3 ligase Smurf1 in HEK293T cells, which were co-transfected with pPPE36-Flag, pMyD88-HA, pSmurf1-Myc, and pUb-Flag. **(B)** Interaction of MyD88 with the E3 ligase Smurf1 in the RAW-PPE36 cells. **(C)** HEK293T cells were transfected with pSmurf1-Myc for 24 h and then transfected with sh-Smurf1 or control shRNA; 12 h later, MyD88 ubiquitination was monitored by immunoprecipitation assays. All data were representative of at least three independent experiments. TCL, total cell lysates; IP, immunoprecipitation; IB, immunoblotting.

Since no analogous functional domain of E3 ligase was predicted in PPE36 protein by searching the UbiBrowser database, we speculated that PPE36 might indirectly promote MyD88 ubiquitination through other ubiquitination ligases. To date, only E3 ligase Smurf1 was shown to not only be involved in Mtb infection (27, 28) but also participate in the MyD88 degradation (28). Therefore, we next investigated the impact of PPE36 on the interaction of MyD88 and Smurf1. We found that compared with other groups, the PPE36-overexpressing group showed more Smurf1 protein binding to MyD88 (**Figure 7A**), indicating that PPE36 obviously promoted the interaction of these two proteins. More importantly, PPE36 efficiently favored the Smurf1-mediated MyD88 ubiquitination (**Figure 7A**). A similar increased Smurf1–MyD88 interaction was also seen in PPE36-stably expressing macrophages (**Figure 7B**). Meanwhile, we found that PPE36-caused MyD88 ubiquitination could be obviously abolished when Smurf1 was downregulated (**Figure 7C**), indicating that Smurf1 was the primary E3 ligase in this process. These data showed that PPE36 promoted Smurf1-mediated ubiquitination and degradation of MyD88.

## Discussion

Mtb is the world’s most successful pathogen. It invades hosts mainly through respiratory tracts, and the first innate immune cells it encounters are alveolar macrophages, which are armed with plenty of immune mechanisms to detect and eliminate intracellular pathogens. After thousands of years of reciprocal evolution with human beings, Mtb has evolved a series of complex immune escape mechanisms that can successfully evade or modulate host immune responses to ensure its successful survival. Studies in murine mycobacterium infection models showed that lung bacterial loads significantly increase during the innate immunity stage, yet stopped growing and achieved the platform when adaptive immune responses initiated (29), further displaying the eximious abilities of mycobacterium to evade host innate immunity.

PE and PPE are two unique families of multigene proteins in Mtb. They encode two major glycine-rich protein families, accounting for nearly 10% of bacterial genomic encoding capability (30). PE/PPE proteins possess a comparatively conserved N-terminal and a highly variable C-terminal, which were conducive to escape the host immune attack (5, 31). In addition, PE/PPE proteins were exclusively restricted to the virulent mycobacterium strains, and this phenomenon was further confirmed by this study. We showed that PPE36 expression was significantly higher in the virulent H37Rv strain and clinical isolates than that in attenuated BCG strains, suggesting that PPE36 might play an essential part in the mycobacterium infection and disease.

Protein localization is extremely critical to its function. However, at present, the location and function of most PE/PPE proteins are still largely unknown. Previous studies reported that some secreted PPE proteins can interact with the host cell surface PRR or enter the host cytoplasm to regulate signaling pathways (17, 32–35). Some studies also showed that cell-membrane- or cell-wall-associated PE/PPE proteins also regulated cell signaling pathways in a variety of forms. For example, the cell-wall-associated protein PPE32 could increase the cytokine secretion by activating endoplasmic reticulum stress (36). PPE44 was reported to promote IL-12 and IL-6 expressions *via* activating NF-κB/Erk1/2/p38 pathways (37). In addition to PPE36, several other PPE family members with similar immune suppression activities have also been reported. For example, PPE10 decreased the expression of IL-1, IL-6, and TNF-α through the linear ubiquitin chain assembly complex (LUBAC) HOIP-NF-κB signaling axis (38); PPE37 inhibited the pro-inflammatory cytokine TNF-α and IL-6 expression in mycobacterium-infected macrophages (39). However, we note that these PPE members mainly acted on the downstream molecules of NF-κB and MAPK inflammatory pathways. In contrast to these studies, herein we found that PPE36 effectively acted upstream of the mycobacterium recognition and PRR-mediated inflammatory pathways, by inhibiting the universal adaptor MyD88 expression at the initial stage of signaling transduction. It is a pity that we did not compare the contribution of other PPE family proteins with similar immune suppression activities. Our study suggested that PPE family members could potently modulate the mycobacterium-induced inflammatory pathways at multiple signaling transduction steps by regulating different molecules. This would help us in better understanding the underlying intricate immune escape mechanisms of mycobacterium tuberculosis. Interestingly, some cleaved products of surface-exposed PPE proteins also possessed the abilities to regulate intracellular signaling pathways. For example, the cleaved C terminal of PPE37 could transport to nuclear and activate the caspase 3-dependent apoptosis pathway (17). In this study, we found that PPE36 could both inhibit NF-κB and MAPK signaling pathways. Correspondingly, BCG ΔPPE36 infection led to the increased inflammatory cytokines (TNF-α, IL-6, and IL-1β) and the reduced lung bacterial loads. We further identified that MyD88 protein was the important target of Mtb PPE36, as the inflammation-suppressive effect was dampened when MyD88 was deficient.

Actually, MyD88 plays an indispensable role during Mtb infection. It was reported that IFN-γ-induced full-scale macrophage activation could only be carried out when MyD88 was present (17). Compared with WT counterparts, MyD88-deficient mice were more sensitive to Mtb infection and more easily progressing to granulomas with massive inflammation and necrosis (40, 41). Our previous study also reported another tuberculosis protein EspR, which could bind to MyD88 protein and inhibit TLR-mediated macrophage inflammation (18). In line with our data, Bandyopadhyay and colleagues (42) showed that Mtb protein sulfotransferase Rv3529c (Stf1) inhibited TLR2-mediated immune response by disrupting the interaction of MyD88 with IRAK1. Of interest, the TcpC protein of virulent *Escherichia coli* also exhibited the ability to regulate the TLR4/NF-κB signaling pathway by acting on MyD88 (24). It seemed that MyD88 might act as a common and key target for diverse bacterial immune-modulating proteins. Herein, we found that PPE36 significantly reduced the intracellular MyD88 expression *via* promoting its ubiquitination and proteasomal degradation, without influencing its gene transcription.

So far, there is no report showing any PPE molecule functioning as an ubiquitin E3 ligase, although their activities as lipase or hydrolase enzyme have been reported (43). Additionally, we did not find out any functional E3 ligase domain in PPE36 with the help of UbiBrowse database previously reported (44); therefore, we speculated that PPE36 may indirectly regulate MyD88 ubiquitination through other E3 ligases. Several E3 ubiquitination ligases have been reported to be involved in MyD88 ubiquitination and degradation, like Smurf1 (18, 28), Nrdp1 (45), and Cbl-b (46). Although all these molecules have been reported to regulate macrophage inflammatory response, only Smurf1 was shown to be involved in Mtb infection. Therefore, we focused on investigating whether PPE36 could affect the interaction of MyD88 and Smurf1. We found that in the presence of PPE36, the Smurf1–MyD88 interaction was significantly increased, and the subsequent MyD88 ubiquitination level was also obviously increased, while when downregulating the Smurf1 expression, the promotion of PPE36 on MyD88 ubiquitination was robustly dampened, suggesting that PPE36 mainly promoted the ubiquitination and degradation of MyD88 through E3 ubiquitin enzyme Smurf1, but whether PPE36 binds directly to Smurf1 needs to be further investigated. In this study, we also found that PPE36 significantly increased K48-dependent, but hardly influence the K63-dependent, poly-ubiquitination on MyD88 protein. In support of our data, Lee and colleagues (18) also reported that TGF-β1-induced MyD88 degradation relied on the K48-linked poly-ubiquitination of MyD88, and of note, in their study, this process was also mediated by the recruitment of E3 ligase Smurf proteins, just like in our experiments. Of note, Smurf1 has been reported as an important factor for restricting Mtb growth, in both murine and human macrophages by promoting autophagy (28). Ironically, here we showed that Mtb PPE36 could in turn exploit this host defense molecule Smurf1 to inhibit host inflammation by promoting MyD88 degradation, further demonstrating the Mtb unparalleled adaptability to its living environment and its powerful gene regulation ability.

Actually, the underlying mechanism of how PPE36 entered the cytoplasm and interacted with signaling molecules is still unclear. However, previous studies may provide some clues for the possible explanation for us. First, studies have shown that virulent Mtb could exist freely in the cytoplasm after escaping from the host cell phagosomes (47), which makes it possible for the Mtb surface protein PPE36 to reach the cytosolic signaling molecules. Although BCG is unable to escape from phagosomes, it could result in membrane-permeable phagosomes, which was permeable to molecules up to 70 kDa (37). Considering that the molecular weight of PPE36 is only 27 kDa, it is very likely to enter cytosols *via* these permeable phagosomes. In addition, PPE37, which is located in the Mtb cell membrane and belongs to the same PPW subfamily as PPE36, has been reported to be cleaved into two fragments (N- and C-terminals) near the transmembrane region by Mtb protease. Moreover, the C-terminal is located in the host cell nucleus and induced apoptosis (17). It is possible that PPE36 might also be detached from bacteria by similar protease cleavage, and then the functional peptides enter the cytoplasm and interact with the signaling molecules. All of these need to be further investigated.

Taken together, our data demonstrated the novel role of Mtb PPE36 in regulating host inflammatory responses and showed that PPE36 could promote the Smurf1-mediated MyD88 ubiquitination and degradation and then dampened host innate immunity and facilitated Mtb survival.

## Data Availability Statement

The original contributions presented in the study are included in the article/**Supplementary Material**. Further inquiries can be directed to the corresponding authors.

## Ethics Statement

The studies involving human participants were reviewed and approved by the Ethics Committee of Affiliated Hospital of Zunyi Medical College. The patients/participants provided their written informed consent to participate in this study. The animal study was reviewed and approved by the Animal Care and Use Committees of Soochow University.

## Author Contributions

ZP performed the literature review, planned the experiments, and performed data interpretation and all of the experiments. YY was involved in the optimization of the experimental protocols. SX and YY supervised the study and performed the data interpretation. All the authors contributed to writing and editing the paper. All authors contributed to the article and approved the submitted version.

## Funding

This work was supported by grants from the National Natural Science Foundation of China (31370894, 31770962, 81970318, and 31670898), the National Science and Technology Key Project (2018ZX10731301-004-003), and the Jiangsu Provincial Innovative Research Team under the Priority Academic Program Development of Jiangsu Higher Education Institutions.

## Conflict of Interest

The authors declare that the research was conducted in the absence of any commercial or financial relationships that could be construed as a potential conflict of interest.

## Publisher’s Note

All claims expressed in this article are solely those of the authors and do not necessarily represent those of their affiliated organizations, or those of the publisher, the editors and the reviewers. Any product that may be evaluated in this article, or claim that may be made by its manufacturer, is not guaranteed or endorsed by the publisher.
