# Supplementary material for: Mycobacterial PPE36 Modulates Host Inflammation by Promoting E3 Ligase Smurf1-Mediated MyD88 Degradation
Source: Front Immunol. 2022 Feb 14;13:690667. doi: 10.3389/fimmu.2022.690667 (PMC8882603; doi:10.3389/fimmu.2022.690667)
Supplement: Supplementary file 1 [file Presentation_1.pptx]

## Slide 1
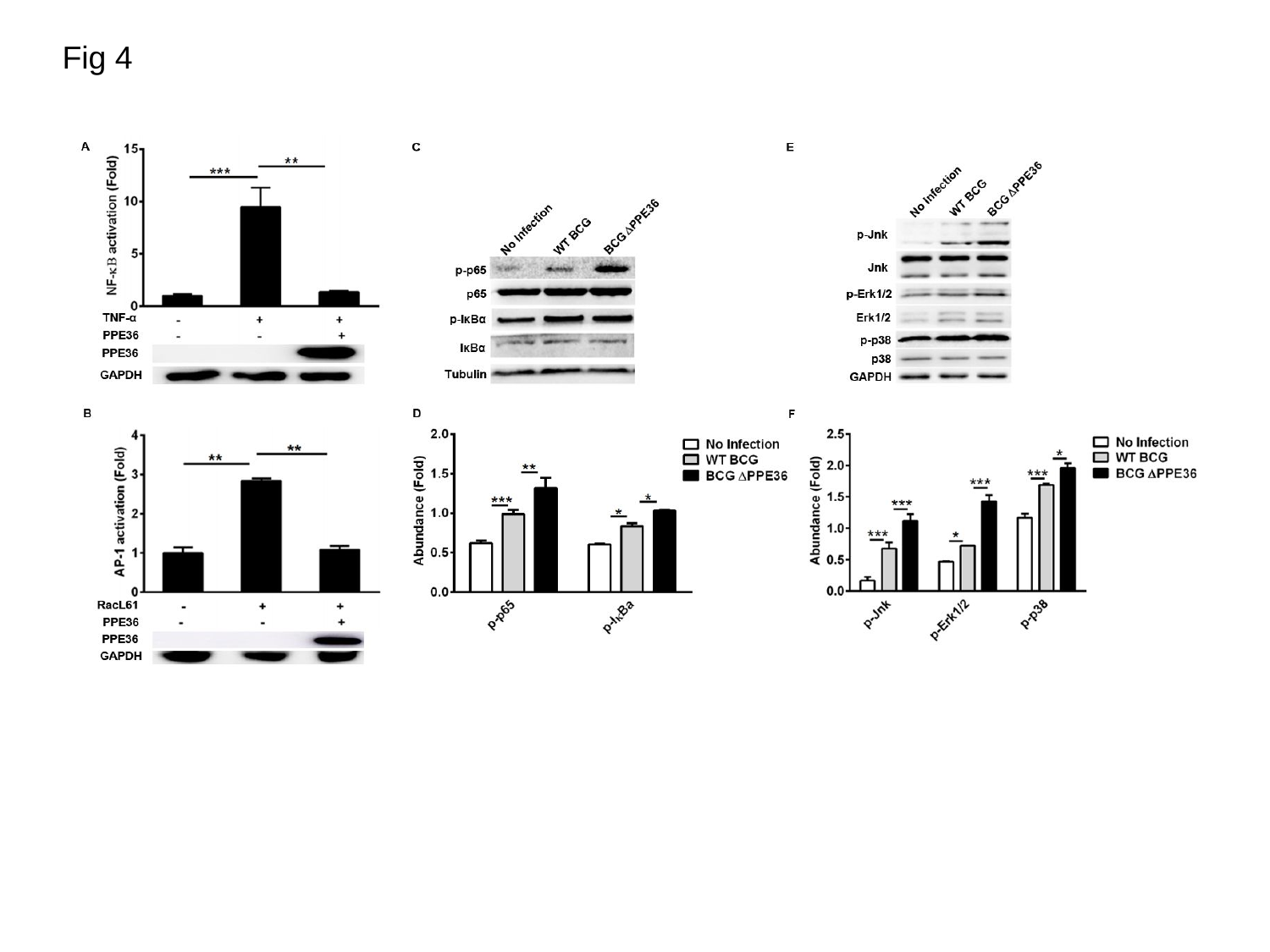

Fig 4

## Slide 2
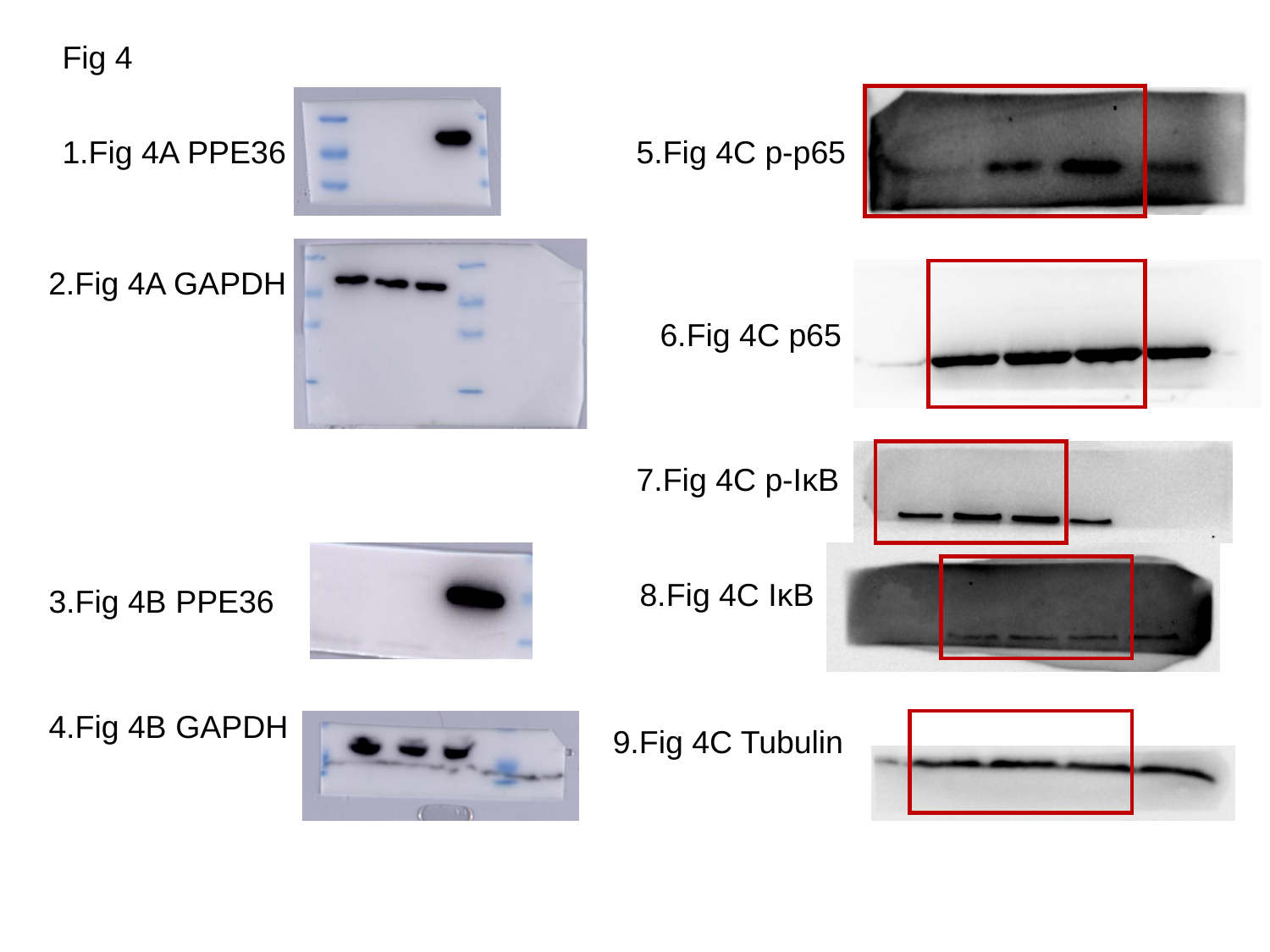

Fig 4
5.Fig 4C p-p65
1.Fig 4A PPE36
2.Fig 4A GAPDH
6.Fig 4C p65
7.Fig 4C p-IκB
8.Fig 4C IκB
3.Fig 4B PPE36
4.Fig 4B GAPDH
9.Fig 4C Tubulin

## Slide 3
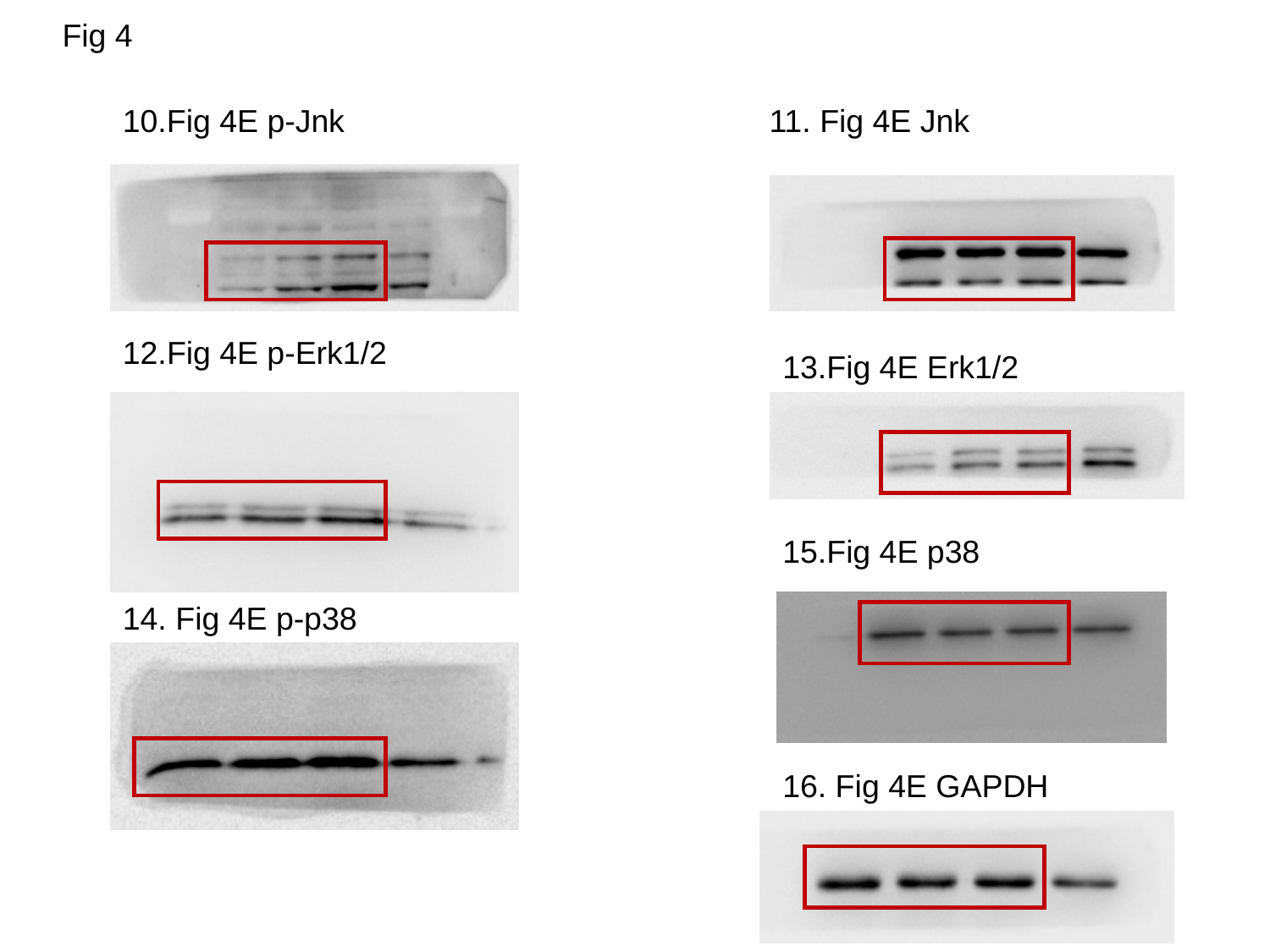

Fig 4
10.Fig 4E p-Jnk
11. Fig 4E Jnk
12.Fig 4E p-Erk1/2
13.Fig 4E Erk1/2
15.Fig 4E p38
14. Fig 4E p-p38
16. Fig 4E GAPDH

## Slide 4
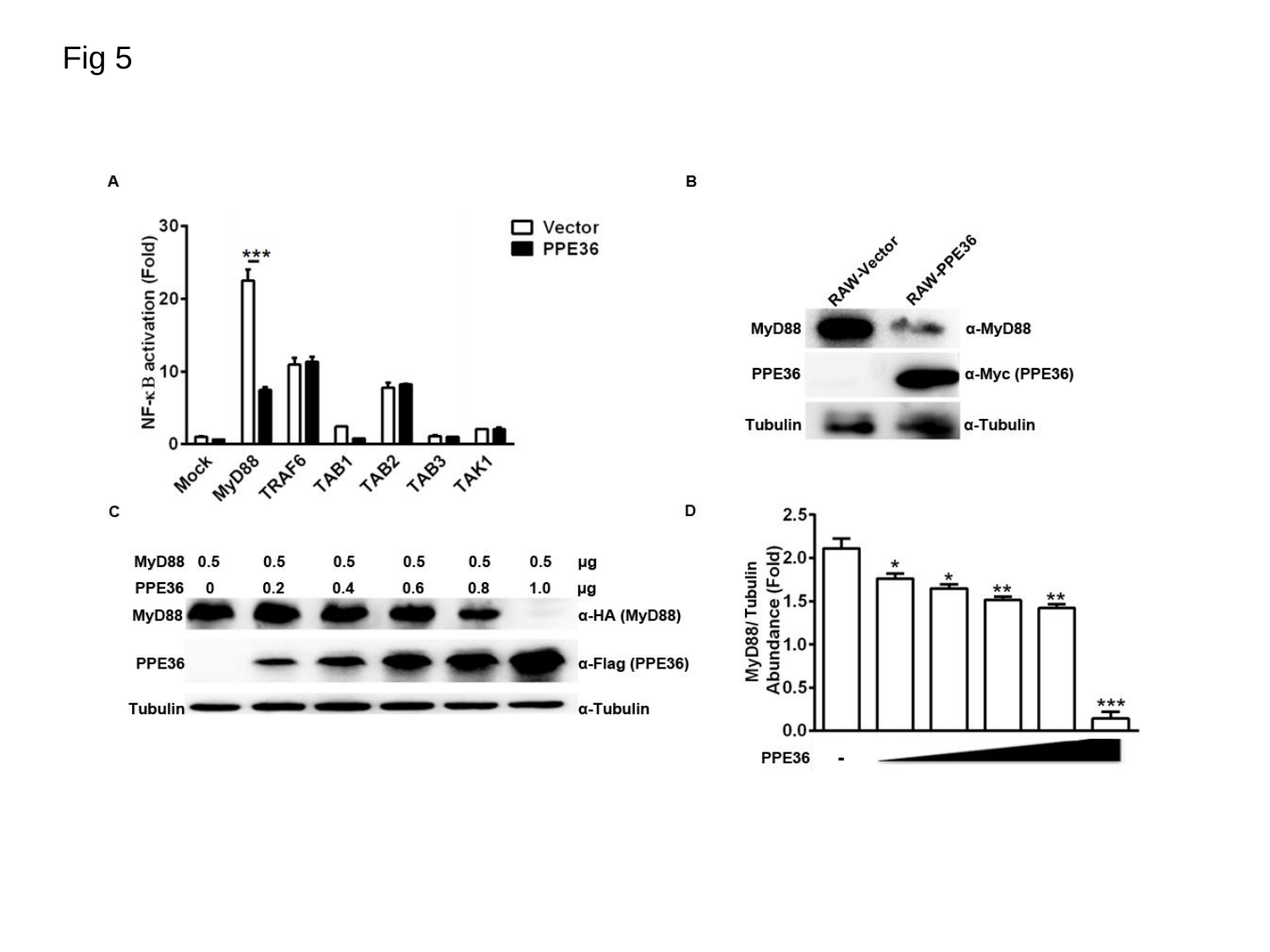

Fig 5

## Slide 5
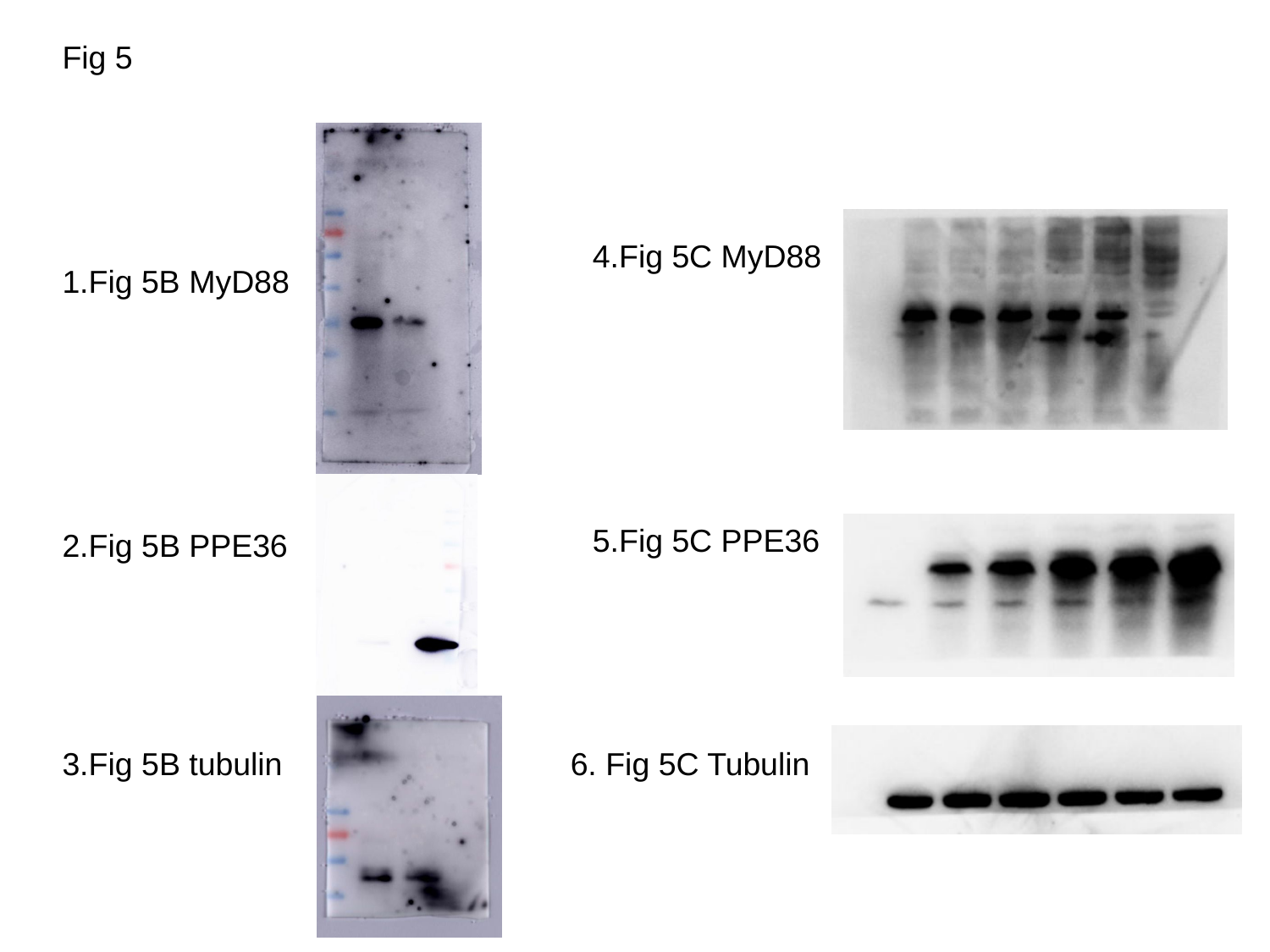

Fig 5
4.Fig 5C MyD88
1.Fig 5B MyD88
5.Fig 5C PPE36
2.Fig 5B PPE36
3.Fig 5B tubulin
6. Fig 5C Tubulin

## Slide 6
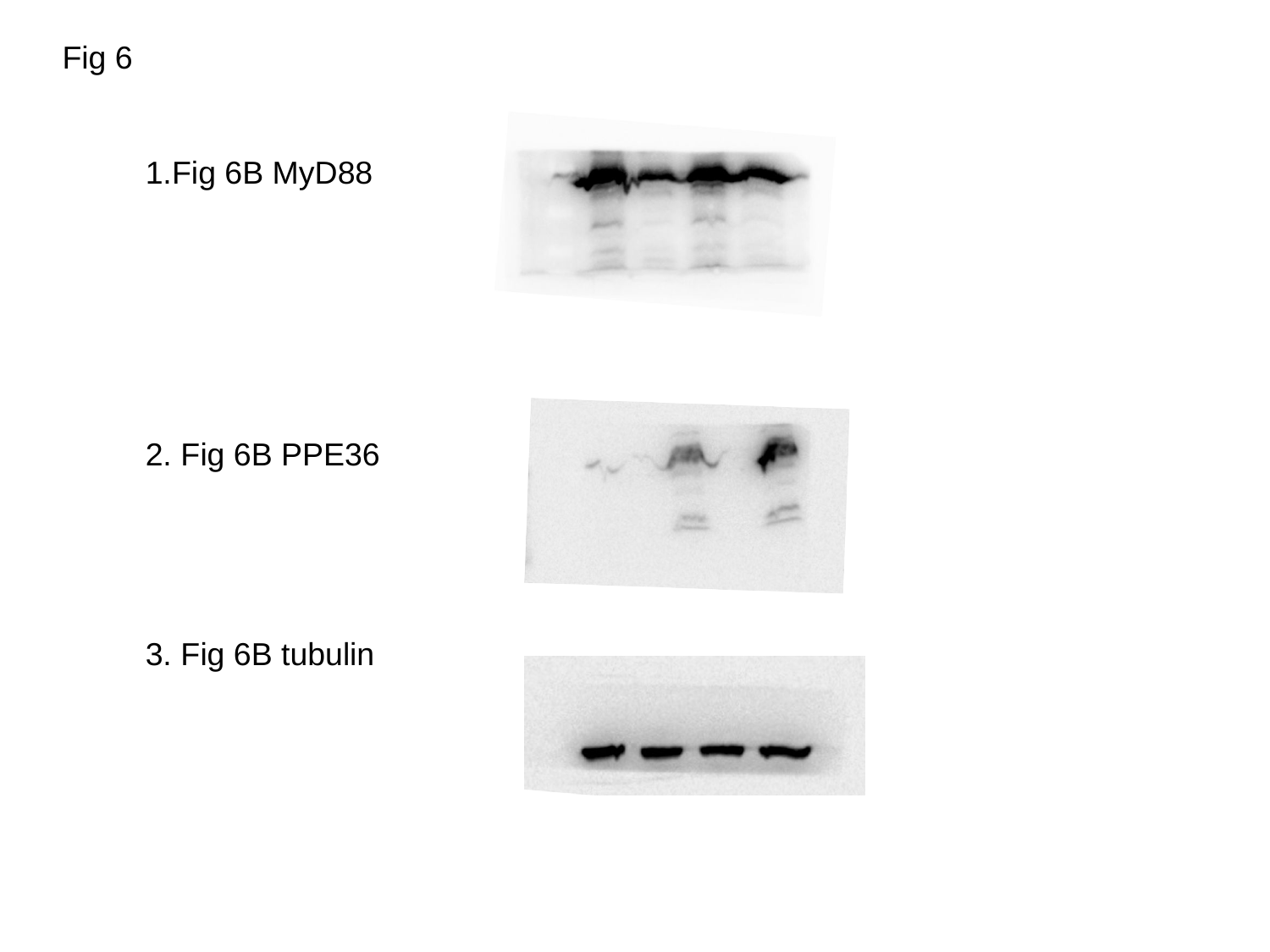

Fig 6
1.Fig 6B MyD88
2. Fig 6B PPE36
3. Fig 6B tubulin

## Slide 7
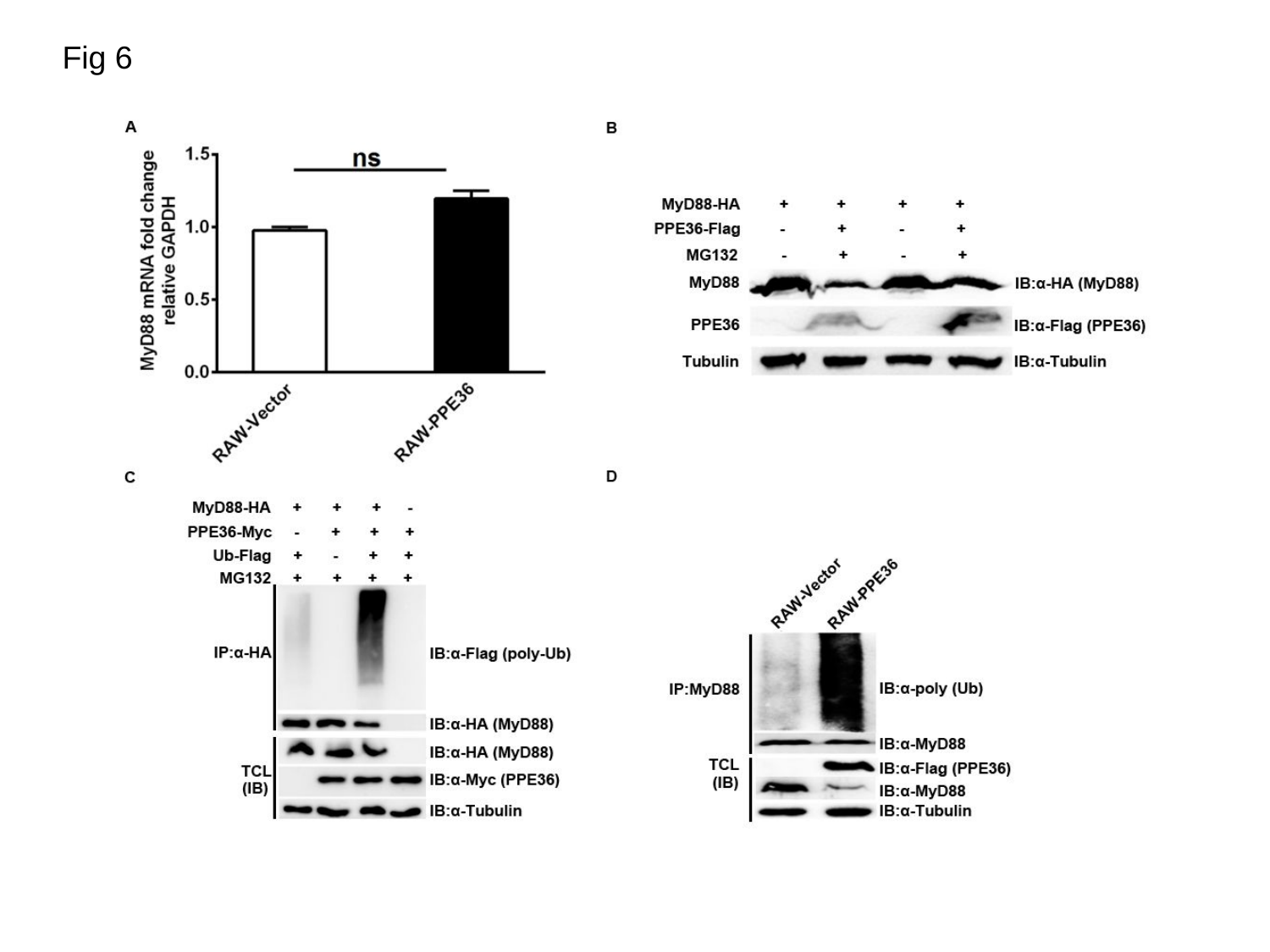

Fig 6

## Slide 8
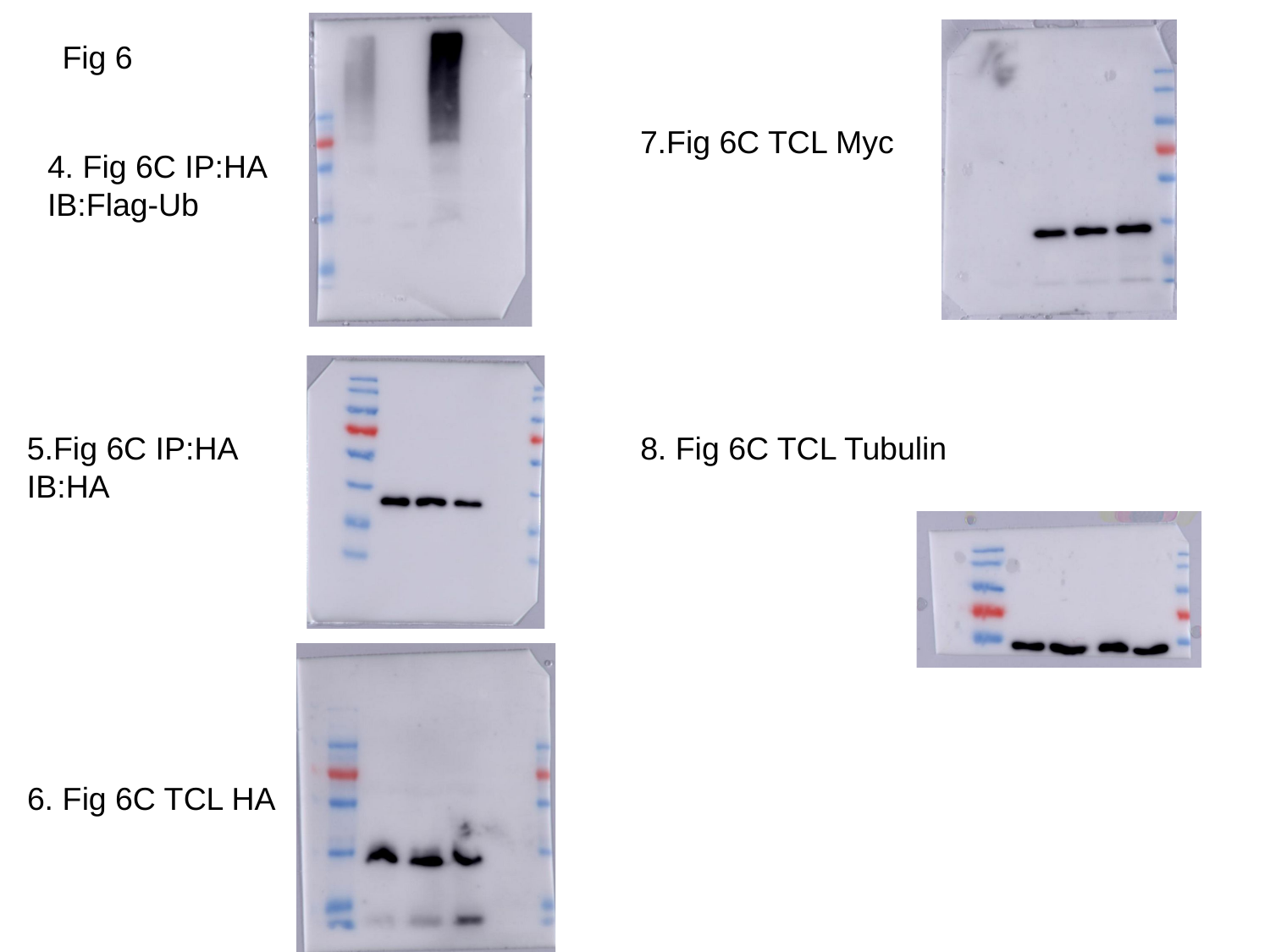

Fig 6
7.Fig 6C TCL Myc
4. Fig 6C IP:HA IB:Flag-Ub
5.Fig 6C IP:HA
IB:HA
8. Fig 6C TCL Tubulin
6. Fig 6C TCL HA

## Slide 9
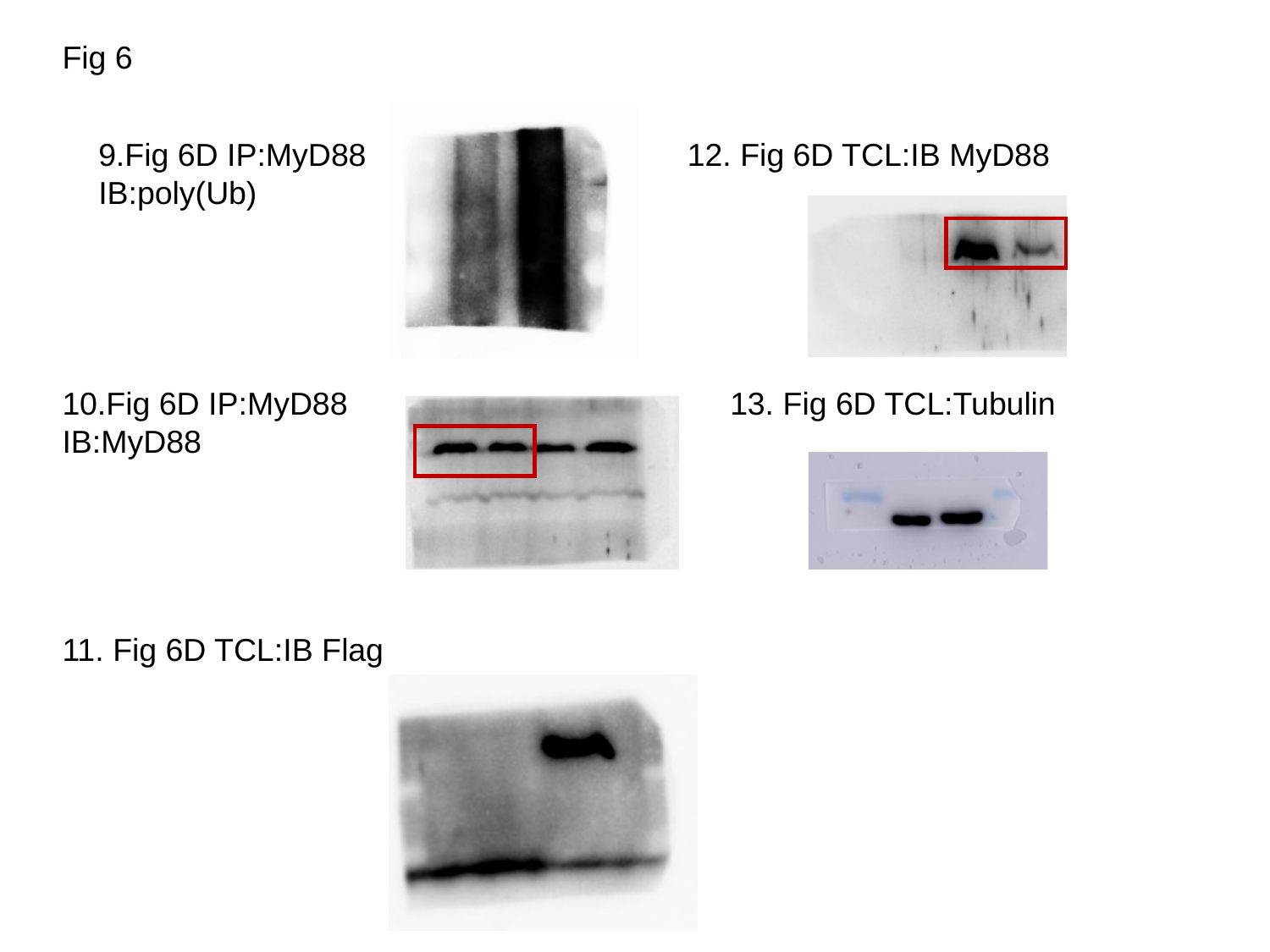

Fig 6
9.Fig 6D IP:MyD88 IB:poly(Ub)
12. Fig 6D TCL:IB MyD88
10.Fig 6D IP:MyD88 IB:MyD88
13. Fig 6D TCL:Tubulin
11. Fig 6D TCL:IB Flag

## Slide 10
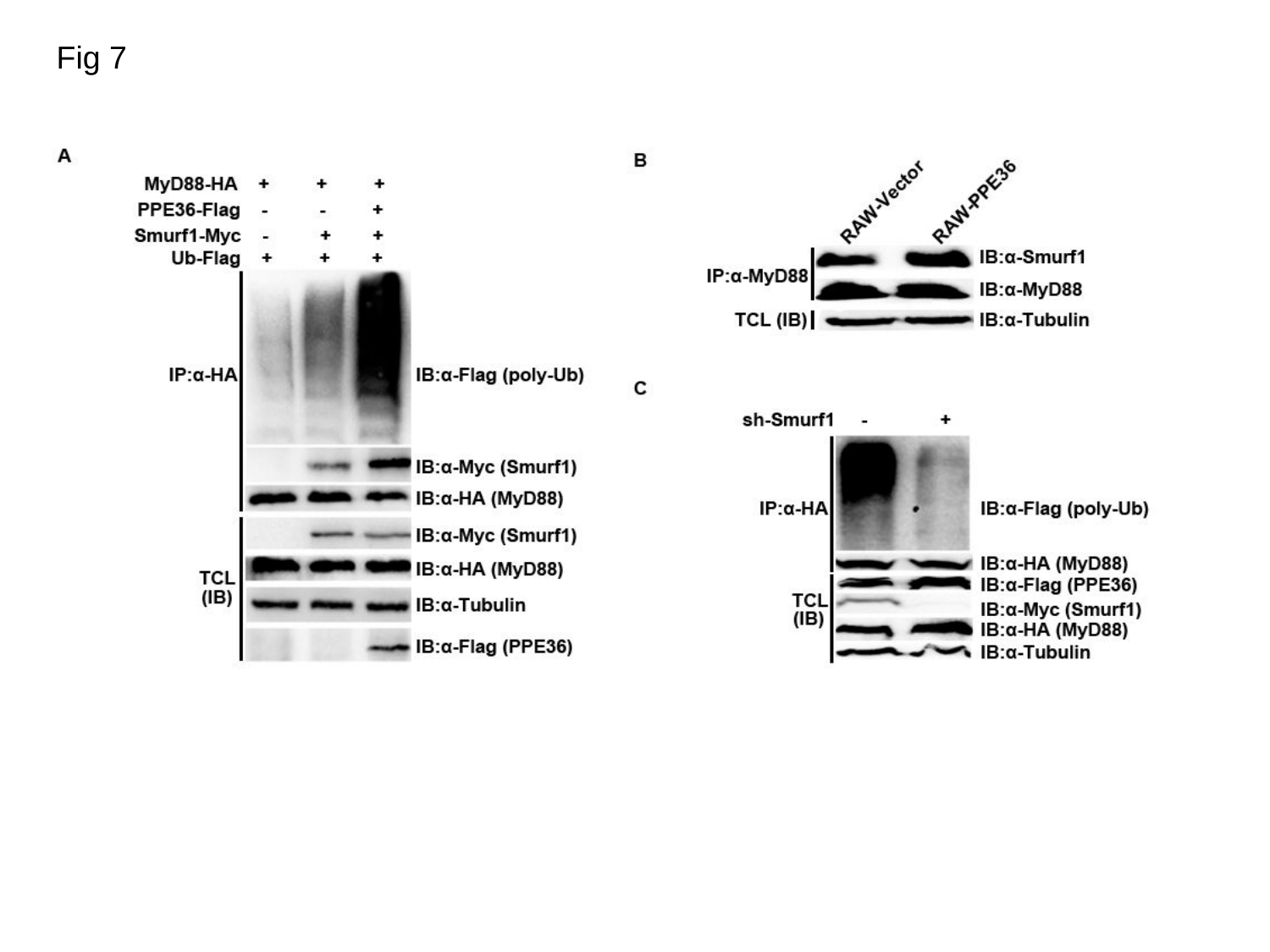

Fig 7

## Slide 11
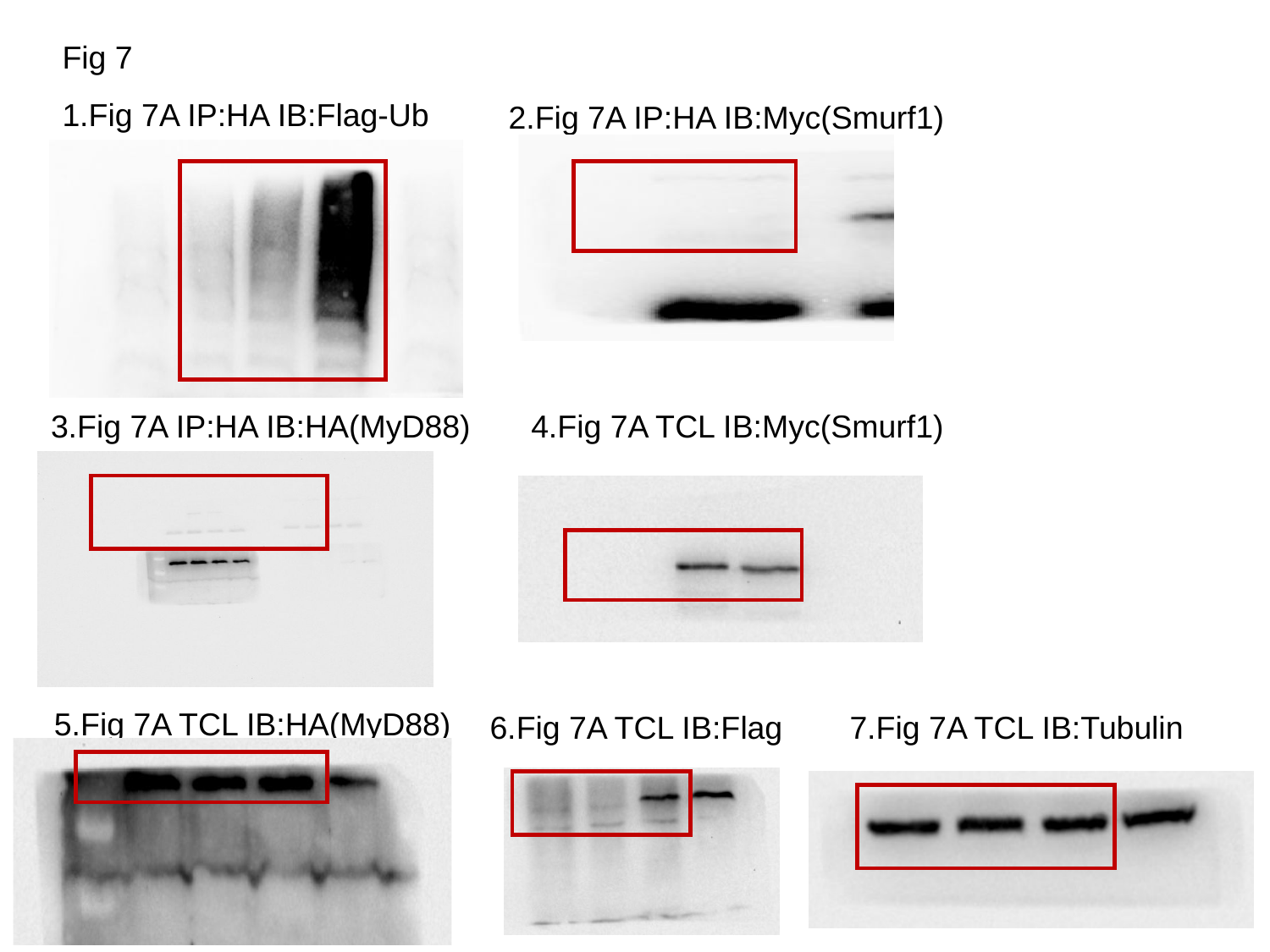

Fig 7
1.Fig 7A IP:HA IB:Flag-Ub
2.Fig 7A IP:HA IB:Myc(Smurf1)
3.Fig 7A IP:HA IB:HA(MyD88)
4.Fig 7A TCL IB:Myc(Smurf1)
5.Fig 7A TCL IB:HA(MyD88)
6.Fig 7A TCL IB:Flag
7.Fig 7A TCL IB:Tubulin

## Slide 12
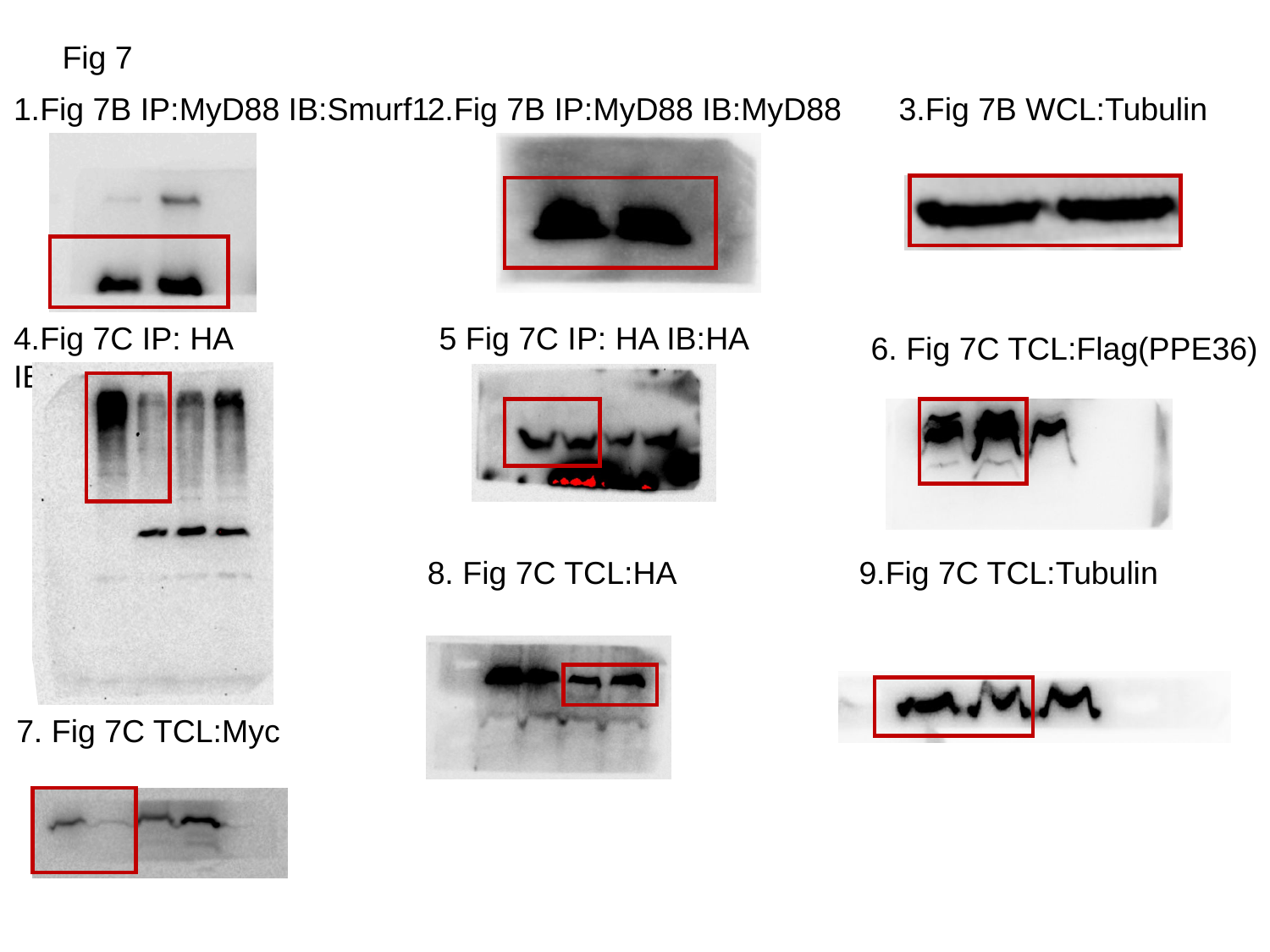

Fig 7
1.Fig 7B IP:MyD88 IB:Smurf1
2.Fig 7B IP:MyD88 IB:MyD88
3.Fig 7B WCL:Tubulin
4.Fig 7C IP: HA IB:Flag(Ub)
5 Fig 7C IP: HA IB:HA
6. Fig 7C TCL:Flag(PPE36)
8. Fig 7C TCL:HA
9.Fig 7C TCL:Tubulin
7. Fig 7C TCL:Myc
